# MIMIC-IV on FHIR: converting a decade of in-patient data into an exchangeable, interoperable format

**DOI:** 10.1093/jamia/ocad002

**Published:** 2023-01-23

**Authors:** Alex M Bennett, Hannes Ulrich, Philip van Damme, Joshua Wiedekopf, Alistair E W Johnson

**Affiliations:** Child Health Evaluative Sciences, The Hospital for Sick Children, Toronto, Ontario, Canada; Institute for Medical Informatics and Statistics, Kiel University and University Hospital Center Schleswig-Holstein, Campus Kiel, Germany; Department of Medical Informatics, Amsterdam UMC Location University of Amsterdam, Amsterdam, The Netherlands; Amsterdam Public Health, Digital Health & Methodology, Amsterdam, The Netherlands; IT Center for Clinical Research, University of Lübeck and University Hospital Center Schleswig-Holstein, Campus Lübeck, Germany; Child Health Evaluative Sciences, The Hospital for Sick Children, Toronto, Ontario, Canada

**Keywords:** fast healthcare interoperability resources, HL7 FHIR, MIMIC-IV, electronic health records, interoperability, open data

## Abstract

**Objective:**

Convert the Medical Information Mart for Intensive Care (MIMIC)-IV database into Health Level 7 Fast Healthcare Interoperability Resources (FHIR). Additionally, generate and publish an openly available demo of the resources, and create a FHIR Implementation Guide to support and clarify the usage of MIMIC-IV on FHIR.

**Materials and Methods:**

FHIR profiles and terminology system of MIMIC-IV were modeled from the base FHIR R4 resources. Data and terminology were reorganized from the relational structure into FHIR according to the profiles. Resources generated were validated for conformance with the FHIR profiles. Finally, FHIR resources were published as newline delimited JSON files and the profiles were packaged into an implementation guide.

**Results:**

The modeling of MIMIC-IV in FHIR resulted in 25 profiles, 2 extensions, 35 ValueSets, and 34 CodeSystems. An implementation guide encompassing the FHIR modeling can be accessed at mimic.mit.edu/fhir/mimic. The generated demo dataset contained 100 patients and over 915 000 resources. The full dataset contained 315 000 patients covering approximately 5 840 000 resources. The final datasets in NDJSON format are accessible on PhysioNet.

**Discussion:**

Our work highlights the challenges and benefits of generating a real-world FHIR store. The challenges arise from terminology mapping and profiling modeling decisions. The benefits come from the extensively validated openly accessible data created as a result of the modeling work.

**Conclusion:**

The newly created MIMIC-IV on FHIR provides one of the first accessible deidentified critical care FHIR datasets. The extensive real-world data found in MIMIC-IV on FHIR will be invaluable for research and the development of healthcare applications.

## BACKGROUND AND SIGNIFICANCE

###  

The digital collection and storage of clinical data have become ubiquitous in healthcare systems around the world. Interoperability of healthcare systems, and in particular the exchange of health data among these systems, has become a major challenge to progress in the field.^1^ Standards are key in solving this challenge as they provide a common language between systems. Health Level 7 Fast Healthcare Interoperability Resources (HL7^®^ FHIR^®^, or simply FHIR) is a standard for data exchange in healthcare.^2^ In just over a decade, FHIR has grown substantially with support from national regulatory efforts and other strategies encouraging wide-spread adoption.^3–5^ National FHIR implementation guides have been created such as US Core,^6^ which provide contextualization of FHIR to unique aspects of a nation’s healthcare system. Ever increasing amounts of patient data are available in FHIR as a result of increased worldwide adoption.^7^ Yet, the use of health data in FHIR stores for research is still in its infancy.^8^ Analysis of data in FHIR will become increasingly important as FHIR stores grow in size and become data sources for operations, research, and predictive modeling.^9^^,^^10^

Data sharing has been a successful mechanism for accelerating research progress in a number of fields. The ImageNet ILSRVC^11^ and related challenges provided an open dataset which enabled broad collaboration toward a shared research aim. The George B. Moody PhysioNet challenges have propelled forward research in physiologic signal processing and data analysis.^12^ Sharing of health data requires caution due to the sensitivity of the underlying content, and the use of synthetic data has been proposed as an alternative to sharing real-world clinical data. Synthea is one approach for generating synthetic data which adopts a data-free model wherein numerous rules were developed by domain experts in order to generate data similar to that encountered during routine clinical practice.^13^ Synthea was used to create SyntheticMass, a FHIR collection of synthetic patients admitted to hospitals in Massachusetts, which has seen broad use in the development and evaluation of FHIR tools.^14–16^ However, synthetic data generated using a data-free model has limited utility in the research context as analyses can only recapitulate known clinical knowledge encoded by domain experts in the generative algorithm. Alternatively, the deidentification of patient data would result in a defacto realistic dataset usable for the generation of new knowledge and algorithms. A number of healthcare datasets have been deidentified and publicly released in the healthcare sector.^17^^,^^18^ To the best of our knowledge, however, no publicly available deidentified datasets currently exist in FHIR.

An ideal candidate to be converted into FHIR is the Medical Information Mart for Intensive Care (MIMIC)-IV database.^19^^,^^20^ MIMIC-IV contains granular data for 73 181 intensive care unit (ICU) stays including patient encounter information, charted observations, laboratory results, microbiology data, medications, and hospital level billing codes. Data from MIMIC has been used in numerous studies ranging from epidemiological investigations to algorithm development.^21^^,^^22^

The objective of this work is to convert the deidentified real-world EHR database, MIMIC-IV,^19^^,^^20^ into FHIR. We included emergency department (ED) data from MIMIC-IV-ED. We further aimed to make a 100-patient subset of this dataset openly available to enable broad reuse of the database. We accomplished these objectives as follows. First, we modeled the MIMIC-IV database as FHIR R4 resources with heavy influence from the US Core Data for Interoperability (USCDI) v1.0 standard.^23^ Second, we generated and published a subset of the MIMIC-IV FHIR resources openly (the MIMIC-IV on FHIR “demo”). Finally, we created a FHIR Implementation Guide (IG) and published the source code for the IG to transparently document our modeling process in accordance with FHIR best practices.

## MATERIALS AND METHODS

**Figure 1.**
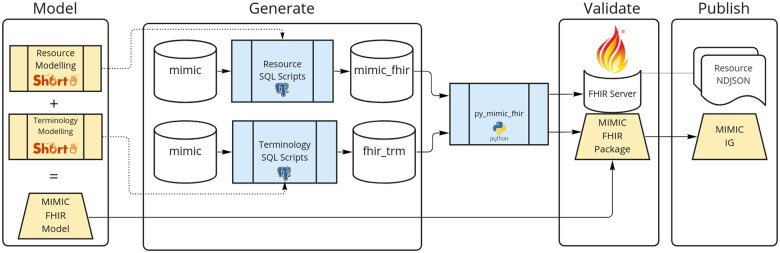
MIMIC-IV to FHIR pipeline. In the model step, the resources and terminology are modeled using FHIR shorthand. The resulting models are then used in the generation step to assist in mapping over the MIMIC tables to MIMIC-FHIR. The generated FHIR resources are then validated for conformance, that is, resources are tested using constraints specified in the generated FHIR Package. A support package, py_mimic_fhir, was created to generate FHIR terminology resources and post resource bundles for validation. In the final step, the resources are published as NDJSON and the MIMIC implementation guide (IG) is published.

The conversion of MIMIC-IV into FHIR was accomplished in 4 stages: *Model*, *Generate*, *Validate*, and *Publish*. *Model*: FHIR profiles for terminology and data present in MIMIC-IV are created from the base FHIR resources. *Generate*: Data and terminology are reorganized from the source relational structure according to the aforementioned FHIR profiles. *Validate*: Resources generated are validated for conformance with the FHIR profiles. *Publish*: FHIR resources are published as newline delimited JSON (NDJSON) files and the MIMIC FHIR Package is distributed as part of the created IG. The process to convert MIMIC-IV into FHIR is summarized in Figure 1.

The intention of our work was to make a canonical set of FHIR profiles that faithfully reproduced the source data. Terminology used in MIMIC-IV was bound to profiles to enable validation of the relevant resources. Extensions were added to the profiles to capture elements not present in the base resources, though this was uncommon. Finally, the naming of the profiles with MIMIC specific names was chosen to make the relation to the source data clear.

We assessed the published resources in terms of number of resources generated, computational time, and storage required. Computational time was calculated for resource generation on a single machine (Lenovo Thinkpad P15s, i7-10510U). Storage size comparisons were completed using operating system tools and PostgresSQL database size functions. We calculated the storage space taken by MIMIC-IV loaded into a database in its original relational structure without indices or constraints. We compared this with the space taken by MIMIC-IV after reorganization of the data into FHIR based resources. Finally, we inserted these resources into a fully featured FHIR server and calculated the storage space taken. Storage comparisons were made for the entire database rather than individual components due to challenges in isolating the impact of individual resource types when loaded into a FHIR server. We further exported data as NDJSON files and compared the compressed and uncompressed file sizes with the original comma separated value (CSV) format used to distribute MIMIC-IV. We compressed the files using gzip v1.10 and zstd v1.4.9 with the default settings on Ubuntu 20.04.

### Model

Resources are the basis of the FHIR specification and function as building blocks for storing information. Resources are intended to cover common use cases in healthcare. Examples of base resources include Patient, Encounter, and Observation. FHIR provides the ability to modify the core resources for specific contexts, a process known as profiling. Profiles specify a set of additional constraints which allow customization of FHIR base resources to local contexts. Related profiles can be bundled into a FHIR package which is typically distributed using a package manager. To aid dissemination, a FHIR implementation guide (IG) can be created which contains all the profiles and resources generated in a FHIR package along with detailed documentation.

The FHIR modeling phase involved creation of a set of profiles, that is, constraints on the base FHIR resources. During the creation of these constraints, we aimed to minimize the number of extensions necessary, minimize the amount of custom conversion code necessary in the generate phase, and ensure all data present in MIMIC-IV was captured in the reformatted FHIR version. Profiles were derived from the base FHIR R4 resources with heavy influence from US Core STU4, as the source hospital of MIMIC-IV is based in the United States. US Core builds upon the USCDI modeling guidelines, and is the gold standard implementation guide for FHIR in the United States.

For the purposes of exposition, we grouped FHIR resources into 1 of 6 categories: administration, organizational, orders, specimen-derived observations, charted observations, and medications. Administration resources included patient details and hospital encounters. Organizational resources included the source hospital information and the description of its constituent care units. Orders included hospital wide provider requests, hospital billed procedures, and hospital billed diagnoses. Specimen-derived observations primarily consisted of laboratory measurements and microbiology tests. Charted observations included those made in the ICU and ED. Finally, medications were sourced from provider orders, an electronic medicine administration record, and pharmacy dispensation records.

MIMIC-IV contained a number of standard and nonstandard terminologies. We captured terminology through the use of CodeSystem and ValueSet resources. In FHIR, a CodeSystem resource captures all possible codes for a given terminology, while a ValueSet resource composes CodeSystem resources and defines the subset of codes considered valid for a given data element. We adopted widespread code systems when possible (eg, SNOMED CT), and otherwise created a new CodeSystem with relevant terms found in the database (eg, drug names). In most situations, a single CodeSystem resource would be used in a single ValueSet resource to bind all observed terms for a given field in MIMIC-IV. For example, the ValueSet for the interpretation of microbiology culture sensitivities bound a single CodeSystem containing all unique terms used for interpretation in the microbiology table. Conversely, specimens are referenced by both microbiology and laboratory data, which use unique terminology for describing the type of specimen. A unique CodeSystem resource was created for laboratory and microbiology specimens independently, and a ValueSet resource was created which bound all codes in the aforementioned 2 CodeSystem resources. This single ValueSet resource was then used to constrain possible terms used in the type field of the Specimen resource.

FHIR modeling was accomplished using the FHIR ShortHand (FSH) language.^24^ FSH allows modellers to define FHIR profiles, terminology, and other artifacts with a simple domain-specific language, instead of creating StructureDefinitions in XML or JSON directly. Profiles were influenced by the US Core STU4 profiles but were not derived from them due to restrictive terminology bindings. Instead profiles specified a parent relationship to a base FHIR R4 resource, and attempted to include all US Core STU4 elements possible. The minimum cardinality of elements in the resources was increased to one if the element had no missing data in MIMIC-IV. Extensions were created if no existing element of the given resource appropriately captured the information in MIMIC-IV. Terminology bindings were added to elements to reference the created MIMIC-IV terminology resources.

As a convention, the naming structure of each profile followed the pattern MimicResourceContext where “Mimic” was a fixed prefix, Resource was the name of the base FHIR resource extended, and Context provided namespacing of resources as necessary.

### Generate

MIMIC-IV was first loaded into a relational database management system (PostgreSQL v11, PostgreSQL Global Development Group) using code from the publicly available MIMIC code repository.^25^ Conversion of MIMIC-IV data into the format stipulated by the developed FHIR profiles was then accomplished through custom Structured Query Language (SQL) scripts. We chose JavaScript Object Notation (JSON) as the serialization format for the FHIR standard, and mapped the content of MIMIC-IV into JSON datatypes within PostgreSQL.

Our process was assessed on both the demo and the full versions of the MIMIC-IV clinical database. The MIMIC-IV Clinical Database Demo is a random sample of 100 patients from the full MIMIC-IV database.^26^ Use of the demo data enabled rapid prototyping with a reasonable comprehensive subset. The full MIMIC-IV data contained 315 000 patients with further details found on the MIMIC-IV PhysioNet page.^19^

Terminology unique to MIMIC-IV was extracted into a set of tables relating each code with a human-readable description of the concept. These terminology sets were mapped to CodeSystem and ValueSet resources in line with the FHIR standard. As the generation of CodeSystem and ValueSet resources followed a consistent pattern, we dynamically generated the resources from the terminology tables using Python. These terminology resources were then embedded into the implementation guide detailed later.

### Validate

FHIR validation was used to ensure proper FHIR structure and consistency with the MIMIC-IV resource and terminology modeling, that is, with the constraints provided in the profiles. Validation was completed using an open-source FHIR server called HAPI-FHIR.^27^ To enable cross-resource validation, resources for an individual patient were ensembled into “bundles” and sent to the FHIR server for validation. When a bundle of resources was submitted to the server, the server then returned either validated FHIR resources or error messages to point to conformance issues. The validation error messages highlighted issues in terminology binding, inter-resource referencing, and improper element mapping. All resources in MIMIC-IV on FHIR were validated against the given profiles using the FHIR server.

### Publish

In addition to publication of the FHIR resources, we created an implementation guide to support understanding of our generated profiles. The implementation guide was made up of 2 main components: the MIMIC FHIR package and the human-readable documentation. The MIMIC FHIR package contained all modeled profiles and custom terminology from MIMIC-IV. This package was used by the FHIR server for validation. The second component was the human-readable documentation describing the MIMIC FHIR artifacts. The official FHIR IG Publisher tool was used to convert the FSH profiles, MIMIC terminology, and markdown documentation into an implementation guide.^28^ Resources were exported from the FHIR server using the bulk export API. The bulk export API is defined in the Bulk Data Access IG which provides a specification for exporting large number of resources from a FHIR server.^29^ We again used HAPI FHIR for bulk export, and wrote data out to NDJSON files.

## RESULTS

**Figure 2.**
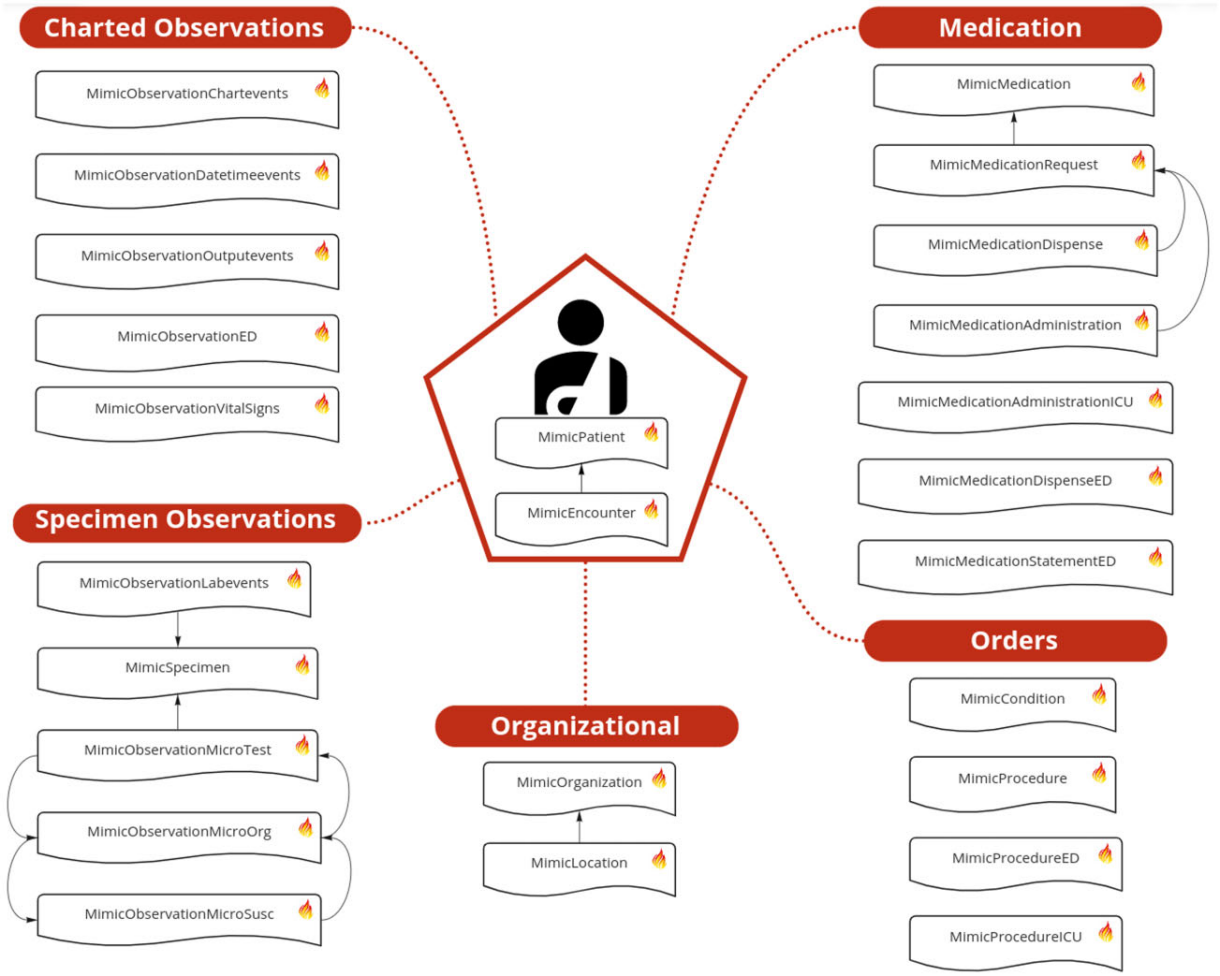
An exemplar patient in MIMIC-IV-on-FHIR with all their associated resources. Administration resources capture the patient and their hospital encounters. Organizational resources capture the source hospital information and the care units. Orders capture hospital wide provider requests, billed procedures, and diagnoses. Specimen observations capture laboratory measurements and microbiology tests. Charted observations capture those made in the intensive care unit (ICU) and emergency department (ED). Medications capture provider orders, electronic medicine administration records, and pharmacy dispensation.

Overall, 25 profiles were derived from the base FHIR R4 resources. MimicPatient and MimicEncounter describe individual patients and encounters and are referenced by nearly all other resources. Figure 2 depicts an exemplar patient in MIMIC-IV-on-FHIR with their resources grouped according to themes Model.

The highest resource count was for charted observations with a median of 2935 resources per patient followed by lab observations with a median count of 452. The typical request, dispense, and administration workflow for in-patient medications was represented by a median of 93 MedicationRequest resource, 91 MedicationDispense resources, and 240 MedicationAdministration resources per patient. A summary of the total and median number of resources per patient is provided in Table 1.

**Table 1.** Resources present in MIMIC-IV-on-FHIR

FHIR category	FHIR profile	Total resources	Demo resources	Median resource count per patient
Administration	MimicEncounter	978 979	637	3
	MimicPatient	315 460	100	1
Organizational	MimicLocation	39	31	–
	MimicOrganization	1	1	–
Orders	MimicCondition	5 953 576	5051	22
	MimicProcedure	704 124	722	4
	MimicProcedureED	2 094 688	1260	10
	MimicProcedureICU	731 788	1468	11
Specimen observation	MimicObservationLabevents	124 342 638	107 727	452.0
	MimicObservationMicroOrg	304 527	338	3
	MimicObservationMicroSusc	1 163 623	1036	14
	MimicObservationMicroTest	2 297 709	1893	8
	MimicSpecimen	15 751 494	12 458	58
Charted observation	MimicObservationChartevents	329 822 254	668 862	2935.0
	MimicObservationDatetimeevents	7 477 876	15 280	56
	MimicObservationED	4 637 088	2742	23
	MimicObservationOutputevents	4 450 049	9362	51
	MimicObservationVitalSigns	10 473 440	6300	50
Medication	MimicMedication	26 536	1794	–
	MimicMedicationAdministration	29 128 087	35 926	240
	MimicMedicationAdministrationICU	9 442 345	20 404	91
	MimicMedicationDispense	13 350 281	14 293	92
	MimicMedicationDispenseED	1 670 590	1082	10
	MimicMedicationRequest	16 217 713	15 225	94
	MimicMedicationStatementED	2 733 573	2411	20
All categories	All profiles	584 068 478	915 281	

*Note*: Profiles created are listed with the total number of resources present in MIMIC-IV v2.0. The demo column provides the resource counts for the 100 patient MIMIC-IV demo dataset. Finally, the median number of resources per patient in the full MIMIC-IV database is listed in the final column.

A total of 34 CodeSystems and 35 ValueSets were generated. The majority of the ValueSets encompass the entire parent CodeSystem. The largest CodeSystems are related to conditions, procedures, and medications. Supplementary Tables S1 and S2 provide details on each CodeSystem and ValueSet generated.

Our comparison of storage size focused on storage required within PostgreSQL for 3 organizations of MIMIC-IV: the original structure, reorganized as FHIR resources with one resource per row, and inserted into HAPI-FHIR. The initial storage space required for initialization of the database was negligible for all organizations (7.9–8.1 MB). Terminology required roughly equivalent space for the original MIMIC-IV structure and the reorganization into FHIR resources, likely due to the minimal overhead imparted by FHIR. Conversely, inserting these resources into HAPI-FHIR resulted in a 12× increase in storage required. For demo resources, the relational structure of MIMIC-IV required the least amount of storage space as anticipated. Conversion into FHIR resulted in an increase of 7.5×, and insertion into HAPI-FHIR required 49× the storage space compared to the original relational structure. Comparing absolute file size outside of the database also highlighted the increased space requirements. When MIMIC-IV-on-FHIR was exported as newline delimited JSON (NDJSON) files, it required 7.5× the storage as the original MIMIC-IV CSV files. Compression with gzip reduced file sizes of the original CSV files by 8.29:1 and of the NDJSON files by 7.36:1. Compression with zstd reduced file sizes of the original CSV files by 8.76:1 and of the NDJSON files by 12.53:1. Nevertheless, the compressed FHIR format NDJSON files are 8.4× larger than the gzip compressed CSV files and 5.2× larger than the zstd compressed CSV files. Table 2 shows the storage size difference between the published MIMIC-IV in PostgresSQL, MIMIC-IV-on-FHIR in PostgresSQL, and MIMIC-IV-on-FHIR in a FHIR Server.

**Table 2.** Comparison of storage requirements between (1) demo data stored in the original relational structure (MIMIC-IV), (2) data reorganized into FHIR (MIMIC-IV-on-FHIR), and (3) the prior FHIR data inserted into a fully featured FHIR server (HAPI-FHIR)

Storage	MIMIC-IV	MIMIC-IV-on-FHIR	HAPI-FHIR
Initial database	7.9	7.9	8.1
+ Terminology	12	13	166
+ Demo data	130	976	6383
Exported file size			
Uncompressed	123.5	918.7	–
Compressed (gzip)	14.9	124.8	–
Compressed (zstd)	14.1	73.3	–

*Note*: Comparisons made in the top 3 rows are done within a PostgreSQL database. The final 3 rows of comparisons are done on exported files. All values presented are in megabytes (MB).

The 915 281 demo resources were generated in PostgresSQL in an elapsed time of 50 seconds. The validation of the demo resources took a further 112 minutes, resulting in 1:07 minutes per patient for validation.

The final MIMIC IG includes 24 profiles, 34 CodeSystems, 37 ValueSets, 2 extensions, and extensive documentation. The implementation guide is available at https://mimic.mit.edu/fhir/mimic.

The generated MIMIC-IV FHIR resources conformant to the MIMIC IG were published as NDJSONs on PhysioNet in 2 forms. A demo of 100 patients with all associated resources is openly available to the public.^30^ A complete version with all data in MIMIC-IV on FHIR will be available to those approved with credentialed access on PhysioNet.

## DISCUSSION

In this work, MIMIC-IV was converted into a set of publicly described and accessible FHIR resources. Researchers, developers, and informaticians will increasingly require expertise with FHIR as national and international initiatives encourage its adoption. The openly available SyntheticMass dataset has seen broad utility in supporting research to date. MIMIC-IV-on-FHIR builds on the success SyntheticMass and other synthetic datasets by providing real-world clinical data in FHIR. The idiosyncracies of electronic health record systems often result in unusual data patterns which are important to capture in research and development.^31^

The extensive detail of FHIR makes it a promising common data model and it was considered as a format for the National Covid Cohort Collaborative (N3C) data sharing initiative which unified information from the i2b2, Observational Medical Outcomes Partnership (OMOP), and TriNetX models.^32–36^. The notable limitation at the time was the lack of bulk export functionality for FHIR, which has since seen rapid development and was successfully used in this work to ingest over a decade of EHR data. An additional common challenge in transforming data into fixed common data models is the need to balance comprehensiveness of the database with ease of use for analysis. Relational database views emerged to support separation of these concerns without harming data integrity. It may be a similar approach is warranted within healthcare wherein the comprehensive FHIR resources, which are unideal for analysis, are used for archival of clinical data. Automated tools would then support transformation of the FHIR resources into more convenient forms for analysis.^37^ We hope the creation of MIMIC-IV-on-FHIR supports further collaborative research into this promising area.

The 4-stage conversion process we adopted could provide a roadmap for future dataset FHIR conversions, although our exact implementation of the approach could be improved. For example, we chose to write SQL due to the ease of using MIMIC-IV within a relational database management system and the maturity of JSON tooling for PostgreSQL. This choice removed our ability to easily enforce strict typing during the development process. Although we surmounted this obstacle through exporting the data to a FHIR server for validation, this process was unideal for development, and alternative approaches which natively enforce typing and constraints could have simplified our work.^38^

Our approach aimed to maximize coverage of MIMIC-IV data while minimizing extensions to base FHIR resources. We considered consolidating FHIR resources into fewer profiles, but decided against this approach as we aimed to represent the source EHR system as accurately as possible in FHIR. For example, the *MimicProcedure* and *MimicProcedureICU* profiles could be merged which would simplify the representation in FHIR at a cost of obscuring the underlying distinct terminology sets and provenance of the data. The modeling process was particularly challenging for medication, microbiology, and encounter resources. We adopted a request-dispensation-administration framework applicable to a number of healthcare contexts. Challenges involved missing data for one or more of these components due to varying levels of digital sophistication for the department providing care and for the time period during which the patient was admitted. Microbiology data was naturally modeled by multiple resources linking the hierarchical relationships between test, sample, isolate, organism, antibiotic, and susceptibility. Nevertheless, the multiple parent-child relationships resulted in a complex set of resources describing what is ostensibly a single observation. Finally, modeling encounters was challenging due to the inclusion of ED data in our work, and the inconsistencies which arise when sourcing redundant information from multiple disconnected systems, in this case the ED information system and the hospital wide electronic health record.

We found validation of FHIR profiles a powerful tool in minimizing errors in our transformation code. To ensure our transformation was correct, we validated all resources generated, a computationally expensive operation. Validation of a large number of resources is an atypical use case and presented challenges to various FHIR servers which we explored. The FHIR team recommends use of validation in development and not in production for efficiency.^39^ Parallelization approaches could be pursued to speed up this process as validation is embarassingly parallel when resources are bundled according to the patient. Numerous cloud vendors offer feature rich FHIR servers which allow for scalable resource ingestion and validation, minimizing the development effort needed for such an approach, albeit at a cost.

The verbose semantics of FHIR provide numerous benefits for interoperability at a cost of storage space. We found representing MIMIC-IV as FHIR resources in a database required 7.5× the storage compared to the original relational structure. This figure grew to almost 50× when storing the data in a fully featured FHIR server backed by the same relational database system. An order of magnitude increase in storage space required is prohibitive for many use cases. Nevertheless, a number of caveats exist for our analysis. First, we stored data within the database systems as JSON, which is primarily an interchange format. Reductions in space are likely possible using other formats currently available such as Parquet, ORC, or Apache Arrow.^40–42^ Future research into this area is warranted as the size of FHIR datasets grow.

Our work has limitations. We did not map to US Core as this would require extensive terminology mapping for compliance with US Core specific ValueSet resources. Terminology present in MIMIC-IV-on-FHIR is local to the source hospital and requires mapping to standard ontologies. This choice was intentional, as we believe mapping our generated resources to US Core using FHIR tools such as ConceptMap resources will result in a more broadly usable approach. We hope to address this in future work. Second, we did not curate any data during our transform, and the database contains a number of biases which occur in data collected during routine clinical practice. As with MIMIC-IV, researchers must analyze the data with caution and awareness of the context within which data were collected. For a subset of resources, elements required a value which was not present in MIMIC-IV. The status element in multiple resources was required but not found, so was imputed as a value of unknown. Finally, we did not map other databases which are linked to MIMIC-IV, including MIMIC-CXR, the MIMIC Waveform Database, and MIMIC-IV-Note.^43^^,^^44^ Processing of alternate modalities of information in FHIR is an emerging area actively pursued by a number of HL7 working groups including the imaging working group. As these standards mature, we hope our work can be extended to cover these alternate modalities of information.

Our work also has a number of strengths. We extensively mapped almost all data within MIMIC-IV to FHIR profiles which are broadly compatible with other implementations including US Core. EHR data within FHIR will provide developers with insight into realities of clinical data collection, and offer researchers a substrate through which to explore this emerging standard. Second, we exhaustively validated all resources within the database, ensuring compliance with our proposed profiles. We hoped to minimize biases in our mapping process through a multi-national collaboration of researchers experienced with health systems in the United States, Canada, Germany, and the Netherlands. Finally, we have made our entire approach open source, allowing the community to investigate and build upon our work.

## CONCLUSIONS

Data sharing is a powerful mechanism for accelerating research progress. The FHIR specification aims to enhance healthcare interoperability, and by extension data sharing, through a unified standard of communication. MIMIC-IV-on-FHIR is a publicly accessible repository of FHIR data pertaining to hospitalizations at a single tertiary academic medical center. A common dataset comprised realistic patient information in FHIR will support a number of research initiatives and the development of healthcare applications.

## Supplementary Material

ocad002_Supplementary_Data

## Data Availability

MIMIC-IV on FHIR is distributed as a collection of newline delimited JSON (NDJSON) files, along with notebooks to support importing the data into FHIR servers including Pathling and HAPI FHIR. A demo of 100 patients for MIMIC-IV on FHIR is openly accessible, while the full version requires credentialed access through PhysioNet.^30^ The NDJSON files can be loaded into any FHIR server compliant with FHIR R4.

## CODE AVAILABILITY

An open source repository, MIMIC-FHIR, was created to store all the components needed to generate and use the MIMIC-FHIR resources. The repository allows for community discussion and collaboration on MIMIC-FHIR: https://github.com/kind-lab/mimic-fhir. Tutorials have been created to walk through the launching of a FHIR server followed by the loading, searching, and aggregation of the MIMIC-IV on FHIR data. The MIMIC Implementation Guide can be found at https://mimic.mit.edu/fhir/mimic
