# Supplementary material for: MIMIC-IV on FHIR: converting a decade of in-patient data into an exchangeable, interoperable format
Source: J Am Med Inform Assoc. 2023 Jan 23;30(4):718–25. doi: 10.1093/jamia/ocad002 (PMC10018258; doi:10.1093/jamia/ocad002)
Supplement: ocad002_Supplementary_Data [file ocad002_supplementary_data.zip › ocad002_Supplementary_Data/tableA1_terminology_captured_codesystem.docx]

| Resource | Category | Terminology | Code Count |
| --- | --- | --- | --- |
| CodeSystem | Administration | AdmissionType | 9 |
|  |  | AdmitSource | 16 |
|  |  | DischargeDisposition | 20 |
|  |  | HcpcsCd | 2202 |
|  |  | Services | 19 |
| CodeSystem | Charted Observation | CharteventsDItems | 2982 |
|  |  | DItems | 434 |
| CodeSystem | General | Units | 683 |
| CodeSystem | Medication | MedAdminCategoryICU | 16 |
|  |  | MedicationEtc | 1208 |
|  |  | MedicationFormularyDrugCd | 4128 |
|  |  | MedicationFrequency | 163 |
|  |  | MedicationGsn | 9430 |
|  |  | MedicationICU | 474 |
|  |  | MedicationMethod | 70 |
|  |  | MedicationMethodICU | 5 |
|  |  | MedicationName | 10198 |
|  |  | MedicationNdc | 5745 |
|  |  | MedicationPoeIV | 2 |
|  |  | MedicationRoute | 106 |
|  |  | MedicationSite | 379 |
| CodeSystem | Orders | DiagnosisICD10 | 18450 |
|  |  | DiagnosisICD9 | 9463 |
|  |  | ProcedureCategory | 14 |
|  |  | ProcedureICD10 | 10233 |
|  |  | ProcedureICD9 | 2555 |
| CodeSystem | Specimen Observation | BodySite | 109 |
|  |  | DLabItems | 1623 |
|  |  | LabFluid | 12 |
|  |  | MicrobiologyAntibiotic | 27 |
|  |  | MicrobiologyInterpretation | 4 |
|  |  | MicrobiologyOrganism | 651 |
|  |  | MicrobiologyTest | 177 |
|  |  | SpecTypeDesc | 104 |
