# Supplementary material for: MIMIC-IV on FHIR: converting a decade of in-patient data into an exchangeable, interoperable format
Source: J Am Med Inform Assoc. 2023 Jan 23;30(4):718–25. doi: 10.1093/jamia/ocad002 (PMC10018258; doi:10.1093/jamia/ocad002)
Supplement: ocad002_Supplementary_Data [file ocad002_supplementary_data.zip › ocad002_Supplementary_Data/tableA2_terminology_captured_valueset.docx]

| Resource | Category | Terminology | Code Count |
| --- | --- | --- | --- |
| ValueSet | Administration | AdmissionType |  |
|  |  | AdmitSource | 16.0 |
|  |  | DischargeDisposition | 20.0 |
|  |  | EncounterType |  |
|  |  | Services | 19.0 |
| ValueSet | Charted Observation | CharteventsDItems | 2982.0 |
|  |  | DatetimeeventsDItems | 188.0 |
|  |  | ObservationCategoryED | 2.0 |
|  |  | ObservationTypeED | 4.0 |
|  |  | ObservationTypeVital | 5.0 |
|  |  | OutputeventsDItems | 77.0 |
|  |  | ProcedureEventsDItems | 169.0 |
|  |  | QuantityUnit | 4.0 |
| ValueSet | General | Units | 683.0 |
| ValueSet | Medication | MedAdminCategoryICU | 16.0 |
|  |  | Medication |  |
|  |  | MedicationEtc | 1208.0 |
|  |  | MedicationFrequency | 163.0 |
|  |  | MedicationGsn | 9430.0 |
|  |  | MedicationMethod | 70.0 |
|  |  | MedicationMethodICU | 5.0 |
|  |  | MedicationRoute | 106.0 |
|  |  | MedicationSite | 379.0 |
| ValueSet | Orders | DiagnosisICD |  |
|  |  | ProcedureCategory | 14.0 |
|  |  | ProcedureICD | 3001.0 |
|  |  | ProcedureTypeED | 2.0 |
| ValueSet | Specimen Observation | BodySite | 109.0 |
|  |  | DLabItems | 1623.0 |
|  |  | LabInterpretation | 1.0 |
|  |  | MicrobiologyAntibiotic | 27.0 |
|  |  | MicrobiologyOrganism | 651.0 |
|  |  | MicrobiologyTest | 177.0 |
|  |  | MicroInterpretation | 4.0 |
|  |  | SpecimenType | 84.0 |
